# Protective Effect of Icariin on the Development of Preimplantation Mouse Embryos against Hydrogen Peroxide-Induced Oxidative Injury

**DOI:** 10.1155/2017/2704532

**Published:** 2017-06-07

**Authors:** Rong Ye, Songhua Xu, Yue Liu, Lili Pang, Xiuli Lian, Yuhuan Zhong, Yang Su, Shie Wang

**Affiliations:** ^1^Department of Human Anatomy, Histology and Embryology, School of Basic Medical Sciences, Fujian Medical University, Fuzhou, Fujian 350122, China; ^2^Anatomy Teaching Office, Basic Medical Science Department, Fujian Health College, Fuzhou, Fujian 350101, China; ^3^Cellular and Developmental Engineering Center, School of Basic Medical Sciences, Fujian Medical University, Fuzhou, Fujian 350122, China

## Abstract

During in vitro cultivation of preimplantation embryos, the balance between ROS production and clearance is disturbed and may lead to incompetent embryos, which might be a main reason of IVF-ET failure. Icariin (ICA) is reported to be active in clearing ROS. The present study aimed to investigate whether ICA could reverse H_2_O_2_ pretreatment-induced mouse preimplantation embryo development arrest and, furthermore, to study the underlying mechanisms by detecting ROS levels, mitochondrial membrane potential (Δ*Ψ*m), and zygotic gene expression. The results showed that, after pretreating mouse 1-cell embryos with 40 *μ*M or 60 *μ*M H_2_O_2_ for 30 min, the developmental rate of each stage embryos decreased obviously. And by adding 40 *μ*M ICA, the developmental arrest of 60 *μ*M H_2_O_2_ pretreated preimplantation embryos was significantly reversed. Immunostaining results showed that, comparing with the control group, ROS levels of H_2_O_2_ pretreated 1-cell embryos were elevated and Δ*Ψ*m levels decreased. By adding ICA, the ROS levels of H_2_O_2_ pretreated 1-cell embryos were decreased and Δ*Ψ*m levels were elevated. Furthermore, RT-qPCR results showed that the addition of ICA reversed the H_2_O_2_-induced downregulation of eIF-1A mRNA expression levels. These results indicate that ICA, when used in appropriate concentration, could decrease ROS levels, increase Δ*Ψ*m levels, and modulate the expression of zygotic gene activation (ZGA) marker gene eIF-1A, and thus promote the development of H_2_O_2_-pretreated mouse preimplantation embryos.

## 1. Introduction

After about thirty years of development, human-assisted reproduction technology, such as in vitro fertilization and embryo transplantation (IVF-ET), has gained great progress. Meanwhile, the lacking of competent early embryos for transplantation usually forces the infertile couples to abandon IVF-ET. Thus, it is of need to improve the in vitro cultivation system, to help the “weak” preimplantation embryos overcome developmental arrest. Of the various harmful factors that may result in developmental blockage, the effects induced by excessive reactive oxygen species (ROS) are considered to be among the most significant ones [1, 2].

Except for anaerobic organisms, oxygen is required by all kinds of animals and plants on earth. Oxygen takes part in important biological processes like metabolism and mitochondrial respiration. While, during the consumption of oxygen, derivatives of oxygen that have active chemical reactivity (and be termed as ROS) are produced, including superoxide anion (O_2_^−^), hydrogen peroxide (H_2_O_2_), hydroxyl free radical (OH^−^), and nitric oxide (NO^−^). When the overproduced ROS break through the defensive walls of the organism, it will result in oxidative injury against the cells: excessive ROS will cause DNA damage, mitochondrial changes, lipid peroxidation, and oxidation modifications of proteins, which will lead to damages against the structures and functions of the cells and finally promote cell death [3].

There are several internal antioxidants that exist around the in vivo developing preimplantation embryos, like follicular and oviduct fluids and various antioxidant enzymes provided by the mother. While during the in vitro cultivation of preimplantation embryos, the lack of maternal antioxidants also contribute to the breaking of ROS production/clearance balance and result in developmental arrest. Thus, antioxidants are widely tested in the cultivation of preimplantation embryos, in order to clear out excessive ROS [4, 5].

The relatively safe and nontoxic properties of natural antioxidants have been revealed by more and more researches in the course of choosing efficient and stable antioxidants. Icariin (ICA) is one type of flavonoids and a main component isolated from the stem leaf of *Epimedium brevicornum*. Flavonoids, which are among the best well-studied natural antioxidants, have been demonstrated to be active in clearing ROS [6]. There are increasing data revealing the efficient functions of ICA in protecting the brain, heart, and other organisms against oxidative injury [7, 8], and a few studies have focused on its antiapoptotic roles in preimplantation embryo cultivation [9]. However, the antioxidative roles of ICA in overcoming the developmental blockage of preimplantation embryos have not been reported, and the molecular mechanisms that ICA may work through should be further revealed.

Mitochondria are reported to be important during mouse preimplantation embryo development [10–12]. Like endoplasmic reticulum, mitochondria take part in maintaining endogenous calcium homeostasis [13]. Besides, mitochondria are able to produce ATP through oxidative phosphorylation, in order to provide energy for spindle movement, cell cycle, and metabolism of the preimplantation embryos [14]. Furthermore, mitochondria also have important roles in mediating cell apoptosis and the transduction of cell signals [14–16]. On the other side, along with these activities of mitochondria, ROS is produced; about 90% of cellular ROS is produced by mitochondria [17]. Moderate level of ROS is reported to be beneficial for the normal biological activities of the cell, for example, differentiation and signal transduction [18], while excessive ROS is harmful to mitochondrial DNA (mtDNA) [3]. During the development from the zygotes through blastocysts, mtDNA does not undergo replication and sustain a constant total amount. Therefore, mtDNA mutation or damage-induced mitochondrial abnormality may have severe impacts on preimplantation embryo development [19, 20].

As the sperms are deprived of nearly all the mitochondria before maturation, thus the mitochondria applied for preimplantation embryo development are maternally derived. Besides mitochondria, there are also other maternal materials that are important for preimplantation development, including maternal-originated proteins and mRNA. These maternal effectors will regulate zygotic gene activation (ZGA) in 1-/2-cell stage mouse embryos [21, 22], and furthermore, the zygotic gene products will promote the completion of maternal-to-zygotic transition during 4-/8-cell stage [23–25]. Therefore, ZGA, which contains a minor wave (ZGA I) at late 1-cell stage and a major wave (ZGA II) at early and mid-to-late 2-cell stage, is considered to be one of the most important biological events after fertilization. ZGA marker genes, like murine endogenous retrovirus-like (MuERV-L), zinc finger and SCAN domain containing 4d (Zscan4d), heat shock protein 70.1 (Hsp70.1), and elongation initiation factor 1A (eIF-1A), are detected in many studies to evaluate the state of preimplantation embryo development [26–28]. MuERV-L is expressed firstly in 1-cell embryos and sustains until blastocyst stage; the inhibition of its expression blocked embryonic development at 4-cell stage [29]. Zscan4d is reported to take part in blastocyst expansion [30]. The expression of both Hsp70.1 and eIF-1A (formerly known as eIF-4C) starts at the first wave of ZGA and correlates with DNA replication [31, 32]. MERVL and Zscan4 have recently been reported to be associated with DNA demethylation in the nuclear [33], while the expression of Hsp70.1 (known as a chaperonin) and eIF-1A may affect the process of posttranscriptional regulation in the cytoplasm; thus, the detection of the combination of these four factors may well reflect the cell state. During preimplantation embryo development, it is still unknown whether ICA could affect mitochondria and ZGA gene expression or not; the objective of the present study is to investigate this possibility.

In the present study, mouse preimplantation embryos were collected and treated with H_2_O_2_ for a short time to induce oxidative injury and then cultured in the medium containing ICA until blastocyst stage to observe the possible reversible effects of ICA; then, ROS and Δ*Ψ*m levels of different groups were detected to preliminary investigate its possible working mechanism. Finally, ZGA marker gene expression was detected to elucidate possible molecular foundations. The results displayed that ICA is able to restore mouse preimplantation embryo development after pretreated with H_2_O_2_, and this reversible effect might correlate with lowered ROS levels and elevated Δ*Ψ*m levels and restore the activation of zygotic gene eIF-1A.

## 2. Materials and Methods

### 2.1. Experimental Animals

Kunming (KM) mice were purchased from SLRC Laboratory Animal Co. Ltd. (Shanghai, China) (male, 4–6 w; female, >8 w) and housed under conditions with controlled temperature (22°C ± 1°C) and light cycle (12 h L + 12 h D) for 5~7 days to adapt to the new environment. Experimental protocols concerning mice handling were under the approval of the Institutional Animal Care and Use Committee (IACUC) of Fujian Medical University.

### 2.2. Main Reagents

The main reagents are the following: Pregnant mare serum gonadotropin (PMSG) (2nd Ningbo Hormone Production Company, Zhejiang, China), Human chorionic gonadotropin (hCG) (Prospec, Israel), M2 medium, ICA, H_2_O_2_ solution and DMSO (Sigma, USA), KSOM medium (Millipore, Germany), TRNzol (Tiangen, Beijing, China), Quick-RNA™ MicroPrep and SYBR® Premix Ex Taq™ (Roche, Swiss), Reverse Transcription Kit and dNTP Mix (Thermo, USA), DAPI and JC-1 (Beyotime, China), and DCFH-DA (Molecular probes, USA).

### 2.3. Mouse 1-Cell Embryo Collection

Female KM mice were intraperitoneally injected with 10 IU PMSG, followed by injecting 6 IU hCG after 46–48 h, and then mated with male KM mice at a 1 : 1 ratio; vaginal plug was checked in the next morning as a sign of fertilization. At 26-27 h post-hCG injection (p-hCG), female mice with vaginal plugs were sacrificed and the oviducts were isolated. One-cell embryos were flushed from the oviducts with M2 medium.

### 2.4. H_2_O_2_ Treatment

KSOM medium of the treated group was made by addition of H_2_O_2_ to a final concentration of 0 *μ*M, 20 *μ*M, 40 *μ*M, 60 *μ*M, or 80 *μ*M and then preequilibrated them for 30 min before use. Results of our preliminary studies (data not shown) showed that short-time treatment of H_2_O_2_ is sufficient to induce obvious ROS elevation in mouse preimplantation embryos and that it requires longer time for ICA to exert its function. Thus, in order to reverse the adverse effects of H_2_O_2_ treatment before it becomes irreversible, to leave time for ICA treatment and to recover 1-cell embryos at the time point within minor ZGA, mouse 1-cells were treated with H_2_O_2_ transiently. For detail, 15~20 mouse 1-cell embryos were placed in the microdrops (33 *μ*L each drop) of KSOM medium supplemented with the different concentrations of H_2_O_2_ and cultured at 37°C, in a 5% CO_2_ incubator for 30 min, then removed from the microdrops, washed in fresh KSOM medium for 3 times, and then cultured in KSOM medium. Continuous observations were made at 45 h, 66 h, and 120 h p-hCG during embryo development.

### 2.5. ICA Treatment of the H_2_O_2_ Pretreated 1-Cell Embryos

Mouse 1-cell embryos were collected, cultured in 60 *μ*M H_2_O_2_ (according to the results of the last experiment) for 30 min, washed for 3 times, and then moved into KSOM medium supplemented with different final concentrations of ICA (0 *μ*M, 10 *μ*M, 20 *μ*M, 40 *μ*M, and 80 *μ*M) for further cultivation until blastocyst stage (the embryos were treated with ICA for 92.5 h, from 27.5 p-hCG to 120 p-hCG). Continuous observations were made at the above mentioned time points until blastocyst stage.

### 2.6. ROS Detection by Using DCFH-DA

2′7′-dichlorofluorescein diacetate (DCFH-DA) is lipophilic and can diffuse freely into the cell, in which it was hydrolyzed to hydrophilic DCFH, and stays inside the cell; DCFH is easily oxidated by ROS and form fluorescent DFC, the intensity of which correlates with cellular ROS levels.

In the present study, mouse 1-cell embryos were collected and separated randomly into 3 groups: (1) KSOM medium cultivation for 30 min and recultured in KSOM (for control); (2) cultured in 60 *μ*M H_2_O_2_ for 30 min and recultured in KSOM (H_2_O_2_ group); (3) 60 *μ*M H_2_O_2_ cultivation for 30 min, washed for 3 times in KSOM medium supplemented with 40 *μ*M ICA (according to the results of the last experiment), and cultured in 40 *μ*M ICA until detection (H_2_O_2_ + ICA group).

After treatment, mouse 1-cell embryos of each group were recovered at 30 h p-hCG and moved into 50 *μ*L KSOM microdrops (15 embryos each drop) containing 20 *μ*M DCFH-DA at 37°C for 20 min. After washing with PVP-PBS, the samples were observed under a fluorescence microscope (Nikon, Japan) at 488 nm to excite the DFC. The images of each 1-cell embryos were analyzed to obtain the gray values.

Notably, DCFH-DA stock solution (100 mM) was made by dissolving 0.02435 g DCFH-DA powder with 0.5 mL DMSO; the working solution was diluted with KSOM medium at a dilution of 1 : 5000 to 20 *μ*M.

### 2.7. Mitochondrial Membrane Potential Detection by Using JC-1

Mitochondrial membrane potential (Δ*Ψ*m) means the potential difference between the two sides of mitochondrial inner membrane. 5,5′,6,6′-Tetrachloro-1,1′,3,3′-tetraethyl-imidacarbocyanine iodide (JC-1) is a widely used lipophilic cationic dye for detecting the relative level of Δ*ψ*m: when the mitochondrial membrane is in a state of low Δ*ψ*m, JC-1 enters the matrix of the mitochondria as monomers (J-monomer) and shows green when excited at 488 nm. On the other hand, when Δ*ψ*m is high, JC-1 will cross the mitochondrial membrane in its aggregate form (J-aggregate) and shows red when excited at 543 nm.

In the present study, mouse 1-cell embryos of each group were collected at 30 h p-hCG and moved into 50 *μ*L KSOM microdrops containing 1 *μ*g/ml JC-1, followed by culturing at 37°C for 20 min. After JC-1 incubation, the samples were washed with PVP-PBS and then detected immediately under the fluorescence microscope. The relative fluorescence intensity of the red and green lights was calculated as an index reflecting mitochondrial activity.

### 2.8. RT-qPCR Analysis

Mouse 1-cell embryos of the 3 groups (40 embryos each group) were recovered separately at 30 h p-hCG, and the total RNA of each group was extracted according to the manual of Quick-RNA MicroPrep kit. RNA concentration and purity were detected by using NanoDrop ND-1000 (NanoDrop, USA). The synthesis of cDNA was performed on a PCR amplifier (AB2720, Gene, USA) according to the manual of Reverse Transcription Kit. Sequence information of the PCR primers used to detect ZGA marker genes, and the reference gene (H2afz) is listed as follows: H2afz (NM_016750, F:5′- GTAAAGCGTATCACCCCTCGT -3′, R:5′- TCAGCGATTTGTGGATGTGT -3′), Zscan4d (NM_001100186, F:5′- CCATCTCATAGTTCTGGTGTGC -3′, R:5′- GCTCCTTAGTCTGCTTTTCTGG -3′), eIF-1A (NM_010120, F:5′- CCAAAGAATAAAGGCAAAGGAG -3′, R:5′- CTCACACCGTCAAAGCACATT -3′), MuERV-L (Y12713, F:5′- CGCACAGCAGCAGTCTATTATC -3′, R:5′- TCTTCTCCTCTTCGGTCAGTTG -3′), Hsp70.1 (NM_010478, F:5′- AAGAGGAAGCACAAGAAGGACA -3′, R:5′-GCGTGATGGATGTGTAGAAGTC -3′). PCR amplification was performed on a real-time PCR amplifier (PikoReal2.2.248, Thermo, USA) by using SYBR Premix Ex Taq with the following cycling protocol: 10 min at 95°C followed by 40 cycles of 15 s at 95°C and 1 min at 60°C. Samples were prepared in triplicate with at least 3 independent repeats.

### 2.9. Statistical Analysis

As H_2_O_2_ may affect the development from 1-cell to 2-cell, the development rates of each stage (2-cell, 4-cell, and blastocyst) were calculated based on the number of 1-cell embryos. SPSS17.0 software was used to perform chi (*χ*^2^)-test. Images of fluorescence microscopy were analyzed by using SmtScape software to obtain gray values. Relative mRNA expression levels were analyzed by 2^(−ΔΔCt)^ method. SPSS17.0 software was used to perform One-way ANOVA. Differences between experimental group and the control group with a *P* value < 0.05 was considered as significant difference.

## 3. Results

### 3.1. Effects of H_2_O_2_ Treatment on the Development of Mouse 1-Cell Embryos

**Figure 1:**
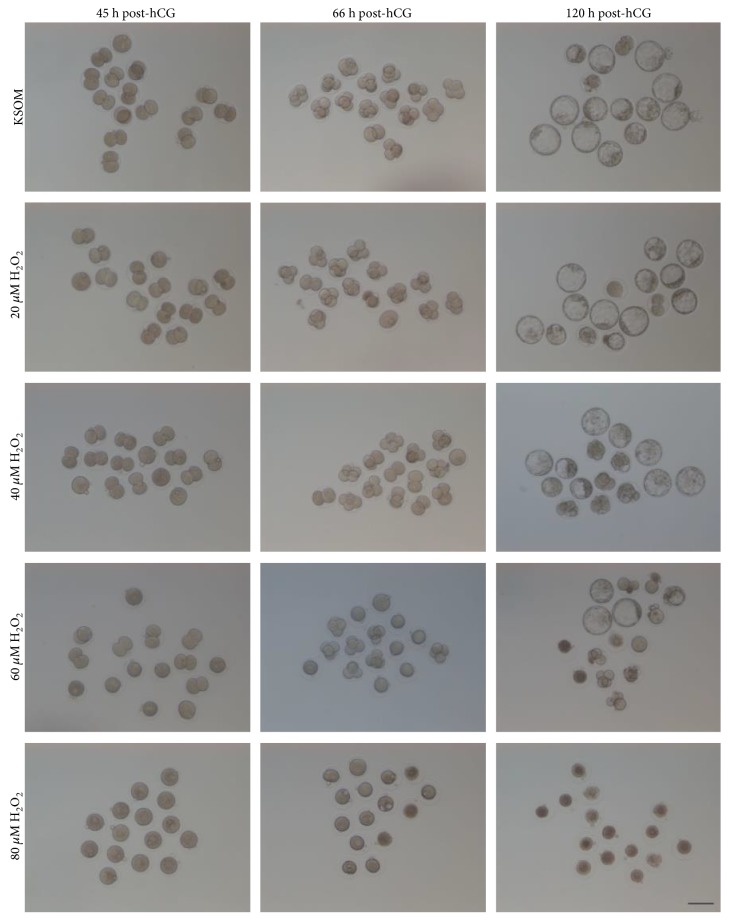
The effects of transient treatment of different concentrations of H_2_O_2_ on the development of KM mouse 1-cell embryos in vitro. Representative images of preimplantation embryos cultured under transient treatment of different concentrations of H_2_O_2_. Zygotes (27 h p-hCG) were pretreated with different concentrations of H_2_O_2_ for 30 min, and then cultured in KSOM until blastocyst (120 h p-hCG). Images of preimplantation embryos were obtained at 2-cell (45 h p-hCG), 4-cell (66 h p-hCG), and blastocyst (120 h p-hCG). In the H_2_O_2_-treated groups, especially the 40 *μ*M and 60 *μ*M groups, developmental retardation was observed. In the 80 *μ*M H_2_O_2_-treated group, preimplantation embryos were arrested in 1-cell stage. Bar = 100 *μ*m.

Comparing with the control group, after treatment of KM mouse 1-cell embryos with different concentrations of H_2_O_2_ for 30 min, the 40 *μ*M group and 60 *μ*M group showed significantly lower (*P* < 0.01) developmental ratios of 2-cell, 4-cell, and blastocyst stage embryos. And 80 *μ*M H_2_O_2_ treatment totally blocked the development from 1-cell to 2-cell stage (Table 1 and Figure 1).

### 3.2. Effects of ICA Treatment on the Development of H_2_O_2_ Pretreated Embryos

**Figure 2:**
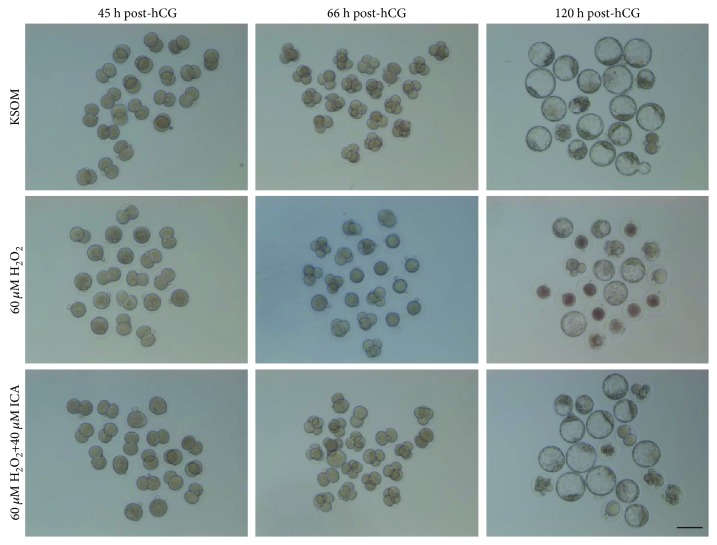
One-cell embryos cultured in the presence or absence of ICA under H_2_O_2_-induced oxidative stress. Zygotes (27 h p-hCG) were pretreated for 30 min with 60 *μ*M H_2_O_2_ and then cultured in ICA until blastocyst stage (120 h p-hCG). Images of preimplantation embryos were obtained at 2-cell (45 h p-hCG), 4-cell (66 h p-hCG), and blastocyst (120 h p-hCG). Restored development of preimplantation embryos was apparent in the ICA-treated group. Bar = 100 *μ*m.

To investigate the ROS clearing functions of ICA in preimplantation embryos, mouse 1-cell embryos were pretreated with 60 *μ*M (a dose that, comparing with 40 *μ*M, may induce higher level of ROS, and, comparing with 80 *μ*M, does not result in severe 1-cell damage) H_2_O_2_ for 30 min and then moved into KSOM medium supplemented with different final concentrations of ICA. The results showed that ICA treatment restored the embryonic development that was hampered by H_2_O_2_ pretreatment. Especially, comparing with the 60 *μ*M H_2_O_2_ group, embryonic development of the 40 *μ*M ICA group was most significantly restored (*P* < 0.01) (Table 2 and Figure 2).

### 3.3. ROS Expression Changes after ICA Treatment

The above results indicate that ICA may have the ability to reverse ROS injury induced by H_2_O_2_ treatment. And the results have confirmed the appropriate concentration of H_2_O_2_ (60 *μ*M) that can block the development of mouse embryos and the most proper concentration of ICA (40 *μ*M) that can reverse the effects of H_2_O_2_ pretreatment.

**Figure 3:**
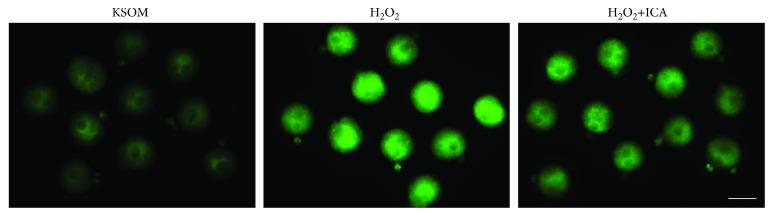
ICA decreases H_2_O_2_-induced intracellular ROS level in KM mouse zygotes. Representative images of ROS levels in zygotes treated with H_2_O_2_ or H_2_O_2_ plus ICA. Intracellular ROS levels were obtained by measuring the intensity of DCF fluorescence (green). Bar = 100 *μ*m.

As we treated 1-cell embryos with H_2_O_2_ transiently for 30 min, it was uncertain whether or not ROS level changes in each group still sustain at late 1-cell (2.5 h after washing) during minor ZGA. Thus, in order to investigate the underlying changes of cellular ROS, we detected the ROS expression levels of each group (KSOM group, H_2_O_2_ group, and H_2_O_2_ + ICA group) by using DCFH-DA. Fluorescence results showed that green fluorescence in the 1-cell embryos distribute diffusely. Comparing with KSOM group, fluorescence intensity in the H_2_O_2_ group was significantly higher (*P* < 0.01). Comparing with H_2_O_2_ group, fluorescence intensity in the H_2_O_2_ + ICA group was significantly lower (*P* < 0.01) (Table 3, Figure 3).

### 3.4. Mitochondrial Membrane Potential (Δ*ψ*m) Changes after ICA Treatment

Mitochondria are important for both ROS production and preimplantation embryonic development. In order to study whether the roles of ICA correlate with mitochondria, we further detected the changes of Δ*ψ*m levels in KSOM group, H_2_O_2_ group, and H_2_O_2_ + ICA group, respectively.

**Figure 4:**
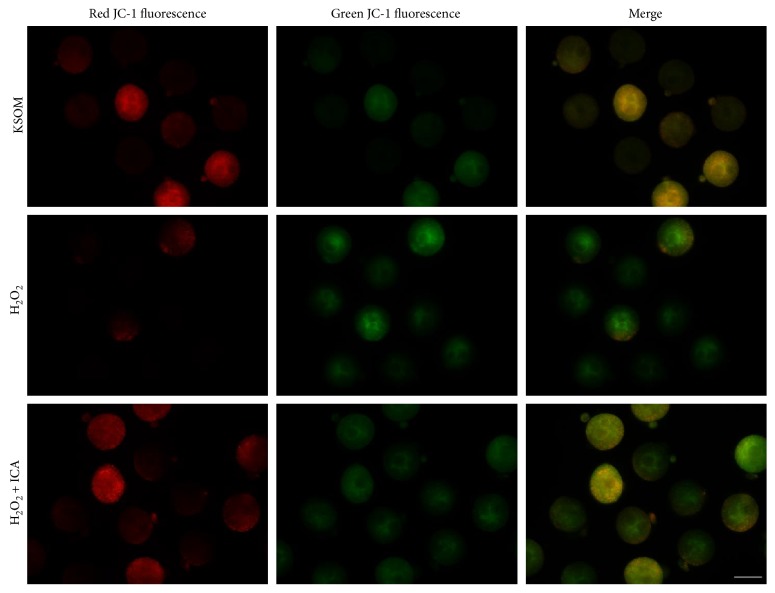
ICA increase H_2_O_2_-induced intracellular mitochondrial membrane potential (Δ*Ψ*m) in KM mouse zygotes. Representative photomicrographs of inner mitochondrial membrane potential (Δ*Ψ*m) in zygotes treated with H_2_O_2_ or H_2_O_2_ plus ICA. Zygotes were stained with JC-1. Red fluorescence represented J-aggregates (high-polarized mitochondria), and green fluorescence represented monomer form of JC-1 (low-polarized mitochondria). Bar = 100 *μ*m.

Fluorescence microscope detection results showed that, after excited, both red and green fluorescence in each group were distributed as speckles. When overlaid, the red fluorescence with the green fluorescence embryos in the KSOM group colored orange obviously (25 embryos colored orange obviously and 10 green). The orange fluorescence intensity of the H_2_O_2_ group was significantly decreased and shown more intense in green (12 embryos colored orange obviously and 24 green), indicating lowered Δ*ψ*m. While that of the H_2_O_2_ + ICA group (20 embryos colored orange obviously and 16 green) was significantly increased than that of the H_2_O_2_ group, indicating restoration of mitochondria activity (Figure 4). The results of statistical analysis confirmed the significance of the differences (*P* < 0.01) (Table 4).

### 3.5. The Changes of mRNA Expression Levels of ZGA Marker Genes after ICA Treatment

Previous studies have shown that ICA could clear excessive ROS and restore mitochondrial function. During the development of 1-cell to 2-cell stage, the activation of zygotic genes is a very critical process and may influence the whole state of the embryo, including the roles of mitochondria. So the mRNA expression of ZGA marker genes was detected to have a glimpse on the molecular mechanisms underlying the effects of ICA treatment.

**Figure 5:**
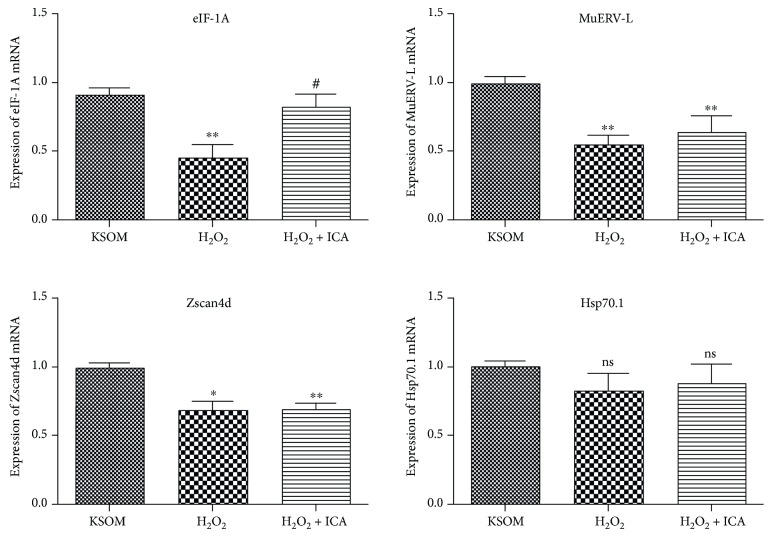
The effects of ICA on the gene expression of Zscan4d, MuERV-L, Hsp70.1, and eIF-1A of KM mouse 1-cell embryos after transient treatment of H_2_O_2_. Differences between the groups were calculated using one-way ANOVA (repetition *n* = 3). Ns: no significant difference (*P* > 0.05), ^∗∗^*P* < 0.01 versus the KSOM group, ^#^*P* < 0.05 versus the H_2_O_2_ group. Bar indicates SEM.

Real-time PCR results showed that, comparing with KSOM group, the expression levels of eIF-1A mRNA decreased obviously in the H_2_O_2_ group (*P* < 0.01); while comparing with the H_2_O_2_ group, eIF-1A mRNA expression levels was increased in the H_2_O_2_ + ICA group (*P* < 0.05). Comparing with KSOM group, the expression levels of both Zscan4d and MuERV-L mRNA in the 1-cell embryos of H_2_O_2_ group and ICA group decreased obviously (*P* < 0.05), while no significant difference of this two genes between H_2_O_2_ group and ICA group was observed (*P* > 0.05). And no significant difference of the expression of Hsp70.1 mRNA levels was observed among these 3 groups (*P* > 0.05) (Figure 5).

## 4. Discussion

Comparing with in vivo development, the in vitro cultured preimplantation embryos would produce more ROS which may induce oxidative injury [34]. It is commonly considered that the ROS levels should be controlled during in vitro cultivation, and many antioxidants have been tested [4, 5]. In the present study, it is shown that appropriate concentration of ICA could reverse the adverse effects that H_2_O_2_ have on mouse 1-cell embryos and significantly increase the developmental ratios of 2-cell, 4-cell, and blastocyst stage embryos. Comparing with H_2_O_2_ group, ICA treatment lowered the ROS levels and increased mitochondrial membrane potential. Besides, mRNA expression of ZGA marker gene eIF-1A was restored after ICA treatment. These results support the hypothesis that ICA could promote in vitro preimplantation embryo development by clearing excessive ROS.

In 2007, Cebral et al. have reported that 50 *μ*M H_2_O_2_ is able to induce mouse preimplantation development arrest at 2-cell stage [35]. Recently, Qian et al. also reported the dose-dependent effects of H_2_O_2_ on preimplantation mouse embryo development and studied the effects of 30 *μ*M H_2_O_2_ on cell cycle and DNA damage regulation [36]. In the present study, we note that 40 *μ*M H_2_O_2_ could significantly affect the development of mouse 1-cells, and 80 *μ*M H_2_O_2_ completely blocked the transition from 1-cell to 2-cell stage (Table 1 and Figure 1). These results indicate that the levels of ROS in the mouse preimplantation embryos should be tightly controlled and demonstrate the importance of testing antioxidants for in vitro cultivation of preimplantation embryos. Interestingly, comparing our results, Cebral et al. showed that the transient treatment of H_2_O_2_ on mouse 2-cell embryos [35] caused more severe effects than that on 1-cell embryos in the present study. Despite the differences in mouse strain and the differences in medium used between these two studies, there may be several possibilities: 1) mouse 2-cell embryos that undergo major ZGA are more sensitive to ROS injury than 1-cell embryos that undergo minor ZGA; 2) there are more maternal factors in 1-cells than in 2-cells and may contain more endogenous antioxidants; 3) mitochondria in one 1-cell embryo are divided equally into two blastomeres of one 2-cell embryo, which reduce its ability to harmonize ROS.

In the present study, we pretreated mouse 1-cell embryos with 60 *μ*M H_2_O_2_ for 30 min and then removed into 40 *μ*M ICA microdrops for further cultivation. Interestingly, the development ratio was restored significantly, and in an ICA dose-dependent way (Table 2 and Figure 2), indicating that ICA is able to clear excessive ROS in the mouse preimplantation embryos. However, when we elevated the concentration of ICA to 80 *μ*M, the developmental ratio did not grow higher, but dropped; this result may suggest that too much ICA might produce adverse factors itself, or that ROS might also have important roles during mouse preimplantation development. Actually, there are increasing data revealing the special “double-edged sword” role of ROS, demonstrating that apart from its effects in induce oxidative injury, appropriate levels of ROS also take important roles in normal biological processes like signal transduction, cell growth, and homeostasis maintenance. It is reported that when increase concentration of O_2_ from 5% to 20%, the in vitro development of bovine 1-cell embryos towards 2-cell was sped up along with elevated ROS levels [37] and that the amount of O_2_ consumption varied dynamically during the development from 1-cell towards blastocyst stage embryos [38]. Thus, the production of ROS is elegantly modulated during this critical course of mammalian development; the disturbance of which might also hamper the preimplantation development. So we chose a relatively appropriate concentration of ICA (40 *μ*M) for further study.

In order to confirm that ICA work through clearing excessive ROS, we separated the 1-cell embryos randomly into 3 groups, KSOM group for control, H_2_O_2_ group, and H_2_O_2_ + ICA group, and detected the changes of ROS levels by using DCFH-DA method. DCFH-DA is able to diffuse into the cell and form fluorescent DCF when oxidized by ROS. The results showed that ROS level was significantly higher in H_2_O_2_ group, lower in H_2_O_2_ + ICA group, and the lowest in KSOM group (Table 3 and Figure 3), supporting that ICA work through clearing ROS. As H_2_O_2_ is one kind of ROS, it is unclear whether the above results reflect the changes of external ROS or endogenously expressed ROS (or the addition of both), although the embryos were washed for 3 times to clear H_2_O_2_ in the medium. Thus, it remains the possibility that ICA does not clear ROS directly, but indirectly through restoring the overall vitality the embryo through its antiapoptotic activities, or through restoring the H_2_O_2_-hampered organelles, like mitochondria, which correlate well with ROS levels and result in increased activity of endogenous antioxidative mechanisms.

At 2014, Zhang et al. used a miR-21 low expression preimplantation embryo model to detect the effects of ICA. Results showed that after ICA treatment, the blastocyst formation rate was increased and the expression levels of miR-21 elevated. Besides, the expression levels of apoptosis factor caspase 3 and PTEN were decreased and that of antiapoptosis factor Bcl-2 was elevated after ICA treatment [9], indicating a role of ICA that correlates with antiapoptosis mechanisms.

During mouse preimplantation embryonic development, the mitochondria have important functions, including programmed cell death, apoptosis, and ATP production. One of the most commonly applied indicators to evaluate mitochondrial activity is mitochondrial membrane potential (Δ*Ψ*m), and the threshold change of which correlates with the level of oxidative phosphorylation, and higher Δ*Ψ*m indicates higher efficiency of ATP production [39]. At 2007, Van Blerkom and Davis performed JC-1 dying to observe the Δ*Ψ*m levels in oocytes; results showed that high oocyte mitochondrial polarity has important roles during fertilization [40]. At 2014, Komatsu et al. reported that at the transition from 1-cell to 2-cell stages, relatively higher Δ*Ψ*m is beneficial for mouse preimplantation embryo development [41]. In the present study, the results showed that the Δ*Ψ*m levels of H_2_O_2_ group were significantly lower than those of the KSOM group and those of the H_2_O_2_ + ICA group were significantly higher than those of the H_2_O_2_ group, indicating restoration of mitochondria activity after ICA treatment (Table 4 and Figure 4). Actually, in samples like nervous cells and brain hippocampus cells, the roles of ICA in protecting mitochondria have been reported [7, 42], but this function of ICA in mouse preimplantation development had not been clearly stated before.

At 2013, Chu et al. treated mouse preimplantation embryos with MEHP, which induced 2-cell blockage and elevated the levels of ROS; by using antioxidant agents, CAT and SOD, the ROS levels were decreased, but it did not reverse the arrest state; RT-PCR results showed that antioxidant treatment could not reverse the changed mRNA expression patterns of ZGA marker genes [27]. The result indicates that at certain conditions, preimplantation embryonic arrest might not mainly relate to ROS disorder and other factors like ZGA failure might have more severe impacts. Results of the present study showed that, comparing with KSOM group, the mRNA expression levels of eIF-1A, Zscan4d, and MuERV-L in H_2_O_2_ group decreased; comparing with the H_2_O_2_ group, the mRNA expression levels of eIF-1A in H_2_O_2_ + ICA group were restored obviously, while other genes did not change significantly (Figure 5). These results suggest that the roles of ICA correlate specifically with certain molecular pathways during ZGA and might thus result in partial restoration of mouse preimplantation embryo development. The start of eIF-1A mRNA expression occurs during 1-cell stage and transiently increases at 2-cell stage [31]; it is considered as an indicator of evaluating preimplantation embryo healthy state and development potential [43]. The roles of eIF-1A might be more diverse than just a marker of ZGA, and the mechanisms that ICA regulates need to be elucidated in our subsequent studies.

In conclusion, the present study showed that ICA is able to reduce ROS levels and reverse the adverse effects of H_2_O_2_ pretreatment on mouse embryo development. ICA could work through modulating mitochondrial activity and regulating the mRNA expression of eIF-1A.

## Figures and Tables

**Table 1:** The effects of transient treatment of different concentrations of H_2_O_2_ on the development of KM mouse 1-cell embryos in vitro.

Group	1-cell embryos	2-cell embryos (%)	4-cell embryos (%)	Blastocysts (%)
KSOM	205	196 (95.61)	190 (92.68)	179 (87.32)
20 *μ*M H_2_O_2_	195	177 (90.77)	170 (87.18)	158 (81.03)
40 *μ*M H_2_O_2_	180	127 (70.56)^∗∗^	109 (60.56)^∗∗^	101 (56.11)^∗∗^
60 *μ*M H_2_O_2_	208	109 (52.40)^∗∗^	88 (42.31)^∗∗^	68 (32.69)^∗∗^
80 *μ*M H_2_O_2_	80	0 (0.00)^∗∗^	0 (0.00)^∗∗^	0 (0.00)^∗∗^

Note: The percentage is based on the numbers of 1-cell embryos. Differences between the groups were calculated using the *χ*^2^-test. ^∗∗^*P* < 0.01 versus the KSOM group.

**Table 2:** The effects of different concentrations of ICA after transient treatment of 60 *μ*M H_2_O_2_ on the development of KM mouse 1-cell embryos in vitro.

Group	1-cell embryos	2-cell embryos (%)	4-cell embryos (%)	Blastocysts (%)
KSOM	119	115 (96.64)	109 (91.60)	103 (86.55)
60 *μ*M H_2_O_2_	108	55 (50.93)	41 (37.96)	31 (28.70)
60 *μ*M H_2_O_2_+ 10 *μ*M ICA	110	57 (51.82)	46 (41.82)	36 (32.73)
60 *μ*M H_2_O_2_+ 20 *μ*M ICA	120	70 (58.33)	59 (49.17)	50 (41.67)^∗^
60 *μ*M H_2_O_2_+ 40 *μ*M ICA	118	94 (79.66)^∗∗^	79 (66.95)^∗∗^	74 (62.71)^∗∗^
60 *μ*M H_2_O_2_+ 80 *μ*M ICA	109	64 (58.72)	51 (46.79)	47 (43.12)^∗^

Note: The percentage is based on the numbers of 1-cell embryos. Differences between the groups were calculated using the *χ*^2^-test. ^∗^*P* < 0.05, ^∗∗^*P* < 0.01 versus the 60 *μ*M H_2_O_2_ group.

**Table 3:** The effects of ICA on the ROS levels of KM mouse 1-cell embryos after transient treatment of H_2_O_2_.

Group	1-cell number	ROS (Mean ± SD)
KSOM	41	7.19 ± 1.60
H_2_O_2_	40	13.44 ± 2.05^∗∗^
H_2_O_2_ + ICA	41	11.08 ± 1.57^∗∗^^##^

Note: Differences between the groups were calculated using the one-way ANOVA. ^∗∗^*P* < 0.01 versus the KSOM group, ^##^*P* < 0.01 versus the H_2_O_2_ group.

**Table 4:** The effects of ICA on the mitochondrial membrane potential of KM mouse 1-cell embryos after transient treatment of H_2_O_2_.

Group	1-cell number	Ratio of J-aggregate to J-monomer staining (Mean ± SD)
KSOM	35	2.72 ± 0.39
H_2_O_2_	36	1.62 ± 0.43^∗∗^
H_2_O_2_ + ICA	36	2.45 ± 0.34^##^

Note: Differences between the groups were calculated using the one-way ANOVA. ^∗∗^*P* < 0.01 versus the KSOM group, ^##^*P* < 0.01 versus the H_2_O_2_ group.
